# Characterizing the expression profile of Dexras1 in human trabecular meshwork cells

**DOI:** 10.1016/j.bbrep.2025.102077

**Published:** 2025-06-10

**Authors:** ChihWei Chen, Jiapeng Han, Luis Sanchez, Judy L. Chen, Jie J. Zheng

**Affiliations:** a Department of Ophthalmology, David Geffen School of Medicine at the University of California, Los Angeles, Los Angeles, CA, USA; b Department of Ecology and Evolutionary Biology, University of California, Los Angeles, Los Angeles, CA, USA; c Department of Molecular, Cell, and Developmental Biology, University of California, Los Angeles, Los Angeles, CA, USA; d The Molecular Bio.logy Institute at the University of California, Los Angeles, Los Angeles, CA, USA

**Keywords:** Trabecular meshwork, Dexamethasone, Dexras1, Glaucoma, Intraocular pressure

## Abstract

Corticosteroids are a mainstay therapy for the treatment of ocular and systemic inflammatory conditions but are associated with a significant risk of intraocular pressure elevation, or ocular hypertension. If intraocular pressure is inadequately controlled, steroid-induced glaucoma may develop, which can result in permanent vision loss and irreversible blindness. Pathological changes akin to fibrosis in the trabecular meshwork, the tissue responsible for intraocular pressure regulation, have been well described and contribute to the development of steroid-induced ocular hypertension and glaucoma. However, the molecular mechanisms driving these fibrosis-like changes in the trabecular meshwork following steroid treatment remain poorly understood. *RASD1* is a gene coding for Dexras1, a small G protein of the Ras family discovered based on its marked induction by the synthetic glucocorticoid dexamethasone. Accumulating evidence points to the role of glucocorticoids in alterations of trabecular meshwork cell morphology, growth, and cell-extracellular matrix interactions. Therefore, we sought to confirm and further characterize how glucocorticoid-induced Dexras1 expression may contribute to glaucoma pathology *in vitro*. In this study, we found that dexamethasone significantly upregulated the expression of Dexras1 in trabecular meshwork cells within 30 min to 1 h post treatment. In addition, we discovered two phenotypes of Dexras1 induction independent of glucocorticoid responsiveness: younger and older donors show significant upregulation of Dexras1, whereas middle-aged donors experience little to no changes in Dexras1 expression after dexamethasone treatment. This age-dependent Dexras1 response may provide a novel explanation for the greater prevalence of steroid-induced glaucoma observed in older and younger populations as opposed to middle-aged populations.

## Highlights

• Steroid glaucoma is related to pathologic changes in the trabecular meshwork.
• Dexras1 is a protein induced in tissues in response to dexamethasone (dex).
• We examined Dexras1 expression in nine human trabecular meshwork cell strains.
• Dex results in time-dependent upregulation of Dexras1 in trabecular meshwork cells.
• We theorize that Dexras1-induced adipogenesis contributes to steroid glaucoma.

Elevated intraocular pressure (IOP) is the most important risk factor for the development of glaucoma. Epidemiologic studies report that there are 60 million people worldwide diagnosed with primary-open angle glaucoma, of which 8.4 million have suffered bilateral blindness [1]. The trabecular meshwork (TM), a structure in the eye that regulates IOP by governing the outflow of aqueous humor, is a crucial structure implicated in glaucomatous pathology. Located in the iridocorneal angle, the TM is a complex connective tissue composed of beams and sheets of extracellular matrix (ECM) surrounded by TM cells, or trabeculocytes. The main responsibility of the TM is to filter cellular debris as a self-cleaning biological filter and to regulate resistance to outflow of aqueous humor [2]. Dysfunction of the TM results in increased outflow resistance that may lead to elevated IOP, which would eventually damage optic nerves and cause vision loss, as characterized by the pathophysiology of primary open-angle glaucoma (POAG) [3].

Dexamethasone (Dex) is a glucocorticoid commonly used to treat ocular inflammation. However, its use in the eye can lead to elevation in IOP, known as steroid-induced ocular hypertension (SIOH). If SIOH is left unrecognized and not appropriately treated, steroid-induced glaucoma (SIG) may develop [4]. Due to the similarity of molecular changes observed in the TM in POAG and SIG, steroid-treated TM cells have been used as an *in vitro* model for studying glaucoma pathology. A key marker for assessing Dex-treated TM cells is the upregulation of myocilin, a protein encoded by the gene *MYOC* that is associated with the glucocorticoid (GC) response in TM cells and is linked to glaucoma pathogenesis [5]. Characteristics of glaucomatous TM include an increase in the deposition of ECM, increased TM cell stiffness, elevated expression level of cross-linked actin networks (CLANs), inhibition of TM cell function, and increased GC responsiveness [6].

Dexras1, encoded by the gene *RASD1*, is a small G protein of the Ras family discovered based on its marked induction by the synthetic GC dexamethasone. The Dexras1 protein is expressed at high concentrations in the brain, and at lower concentrations in several organs throughout the body, including the heart, kidney, liver, skeletal muscle, and pancreas [7], and its functions are multifaceted and complex. Dexras1 has been reported to play a major role in circadian rhythm and cancer by mediating the mitogen-activated protein kinase (MAPK) signaling pathway that may result in photic response and/or abnormal cell proliferation. It is also associated with systemic cardiovascular disease and Dopamine (D1 and D2) receptor-mediated behaviors, such as locomotion activity [7]. In addition, Dexras1 is known to play a part in neuronal diseases as research has reported that S-nitrosylation of Dexras1 contributes to Aβ neurotoxicity, a key contributor to Alzheimer's Disease in which the peptide Aβ deposits in brain tissue. Neurons engineered with mutant Dexras1 are found to be protected from Aβ-induced neural impairments [8].

The role of Dexras1 in the eye is not well-understood, but the rapid induction of Dexras1 by dexamethasone in other tissues suggests that this gene plays a key role in steroid-induced effects [5]. Given the known effects of GCs on the TM and their direct relationship with Dexras1, we aimed to explore GC-induced Dexras1 expression in human TM cells. We established a robust protocol to culture TM cells from fresh human TM tissues derived from unused corneal tissues after transplantation at the UCLA Stein Eye Institute. To ensure the quality of the TM, the death-to-preservation time is < 12 h, and the maximum death-to-TM cell isolation is 7 days. All donors provided informed consent for using their tissues in research. This protocol complies with the requirements of the Declaration of Helsinki, and it has been registered at the UCLA Institutional Review Board (UCLA IRB#: 15–001886) and approved by the UCLA Institutional Biosafety Committee.

Following our established protocol [[9], [10], [11]], corneoscleral rims were first quartered along the anatomical transverse and sagittal planes. Under a Leica MEB 126 dissecting microscope, fine-tip forceps were used to extract the TM tissue from the anterior portion of each rim. The isolated TM pieces were placed individually into six-well plates pre-coated with 0.5 % filtered gelatin. Cells were cultured in Dulbecco's Modified Eagle's Medium (DMEM) with Gluta-MAX containing 1 g/L d-glucose, 110 mg/L sodium pyruvate, 10 % fetal bovine serum (FBS), and 1 % (100 × ) antibiotic-antimycotic or penicillin-streptomycin. The medium was replaced every 2–3 days.

Once cells reached confluence, they were passaged to a fresh gelatin-coated plate. To passage, the spent medium was removed, and the cells were rinsed three times with Dulbecco's phosphate-buffered saline (DPBS). Sufficient 0.25 % trypsin containing ethylenediaminetetraacetic acid (EDTA) was added to cover the cells, and they were incubated at 37 °C and 5 % CO_2_ for 5 min. The trypsin was then neutralized by adding medium, and the cells were resuspended and transferred to a new gelatin-coated container. After 24 h of attachment, routine culturing procedures were resumed.

In this study, we examined the effect of Dex on *RASD1* expression in TM cells from nine donors aged 18 to 66 (Table S1). Donor ages ranged from young (Donor 1: 19 years old; Donor 2: 18 years old, female) to elderly (Donor 3: 59 years old; Donor 4: 61 years old; Donor 5: 66 years old, male). Middle-aged donors were 38 (Donor 6, male), 40 (Donor 7), 55 (Donor 8), and 50 (Donor 9) years old. All cultured TM cells had the standard TM cell morphology matching previous descriptions [12].

**Fig. 1:**
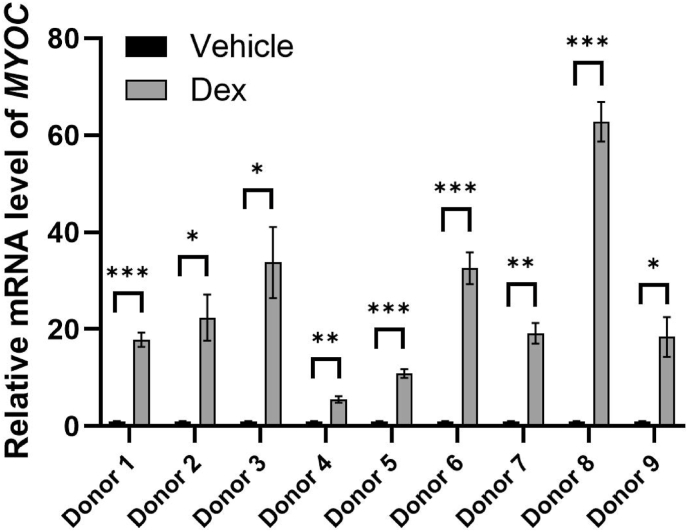
**Characterization of myocilin expression in 9 human TM cell strains treated with vehicle control versus dexamethasone.***MYOC* expression increased in 9 HTM cell strains following 5-day 100 nM Dex treatment. The mRNA fold-changes were GAPDH-normalized. Each measurement was done in triplicate. Error bars represent means ± SEM in biological replicates, except for Donor 8 and Donor 9, where biological replicates were not available due to limited cell counts so technical replicates were used instead for statistical analysis. ∗*P* < 0.05; ∗∗*P* < 0.01; ∗∗∗*P* < 0.001.

As elevated myocilin expression after Dex treatment is a hallmark of TM cells [5], to validate the nine isolated TM cell strains, we examined the expression levels of MYOC mRNA in these cells after Dex treatment as we previously described [13]. Dex treatment of passage 2 TM cells began once the cells reached confluence in six-well plates. Three wells were exposed to 100 nM Dex dissolved in dimethyl sulfoxide (DMSO), while the remaining three received 0.05 % (v/v) DMSO as a vehicle control. The medium containing Dex or vehicle was replaced once over the five-day treatment period. After this period, total cellular RNA was isolated from the TM cells using the Qiagen RNeasy Mini Kit (Qiagen, Valencia, CA) according to the manufacturer's protocol. RNA concentration was measured with a NanoDrop 2000 (ThermoFisher, Waltham, MA), and its quality was evaluated based on the 260/280 nm absorbance ratio. Quantitative PCR was performed using the qScript-XLT 1-Step RT-qPCR kit from Quanta Biosciences (Beverly, MA) following the manufacturer's instructions on a Realplex 2 system (Eppendorf, Hauppauge, NY), employing TaqMan primers for human GAPDH (Hs02758991_g1) and human MYOC (Hs00165345_m1) from ThermoFisher Scientific (Canoga Park, CA). Finally, GAPDH-normalized MYOC fold changes for each TM strain were calculated using the Delta-Delta Ct method. All data are represented as the mean ± standard error of mean (SEM). A two-tailed unpaired Student's *t*-test with unequal variance was used to determine statistical differences with a significance level of *P* < 0 0.05. We found that the TM cells from all nine donors showed notable upregulation of *MYOC* mRNA via RT-qPCR following a 5-day 100 nM Dex treatment (Fig. 1), confirming all nine cell strains are steroid-responsive TM cells.

We then examined Dexras1 expression levels induced by Dex treatment in these nine TM cell strains. Since the expression of Dexras1 has been observed to increase within hours after Dex treatment in other cell types, we decided to examine the expression levels of Dexras1 after Dex treatment in the time frame of hours. Because the cells at passage two were used for steroid-response validation, cells from the third passage were used in most of these studies. The cells were treated with either DMSO vehicle or 100 nM Dex for 0, 0.5, 1, 2, 3, or more hours according to the experiment plan. Then, total cellular RNAs were extracted, and RT-qPCRs were conducted as described in the earlier section; the primers for human RASD1 (Hs02568415_s1) were also obtained from ThermoFisher.

**Fig. 2:**
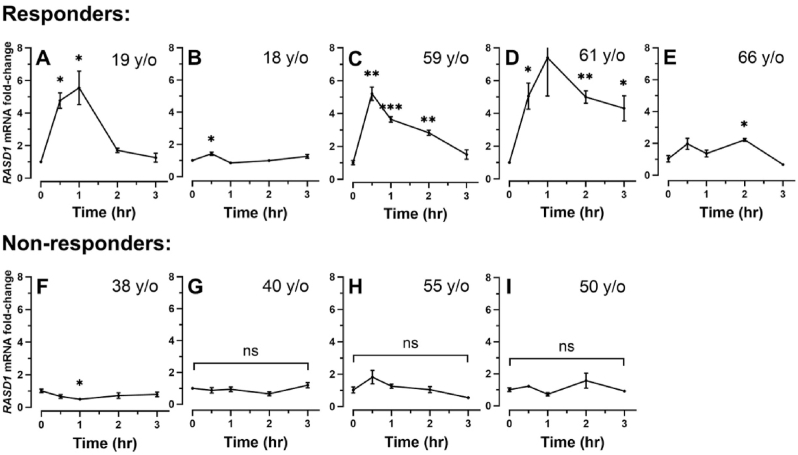
**Dexamethasone regulates *RASD1* expression in a donor-dependent manner.***RASD1* expression is rapidly upregulated in Donor 1–5 within 30 min to 1 h of 100 nM Dex treatment and decreases thereafter (panels A–E). In Donor 6–9, *RASD1* expression remains largely unaltered within 0–3 h of Dex treatment (panels F–I). Data points show GAPDH-normalized Dexras1 mRNA fold-change after 0, 0.5-, 1-, 2-, and 3-h treatment with Vehicle control or 100 nM Dex at passage 3 (except for Donor 2, which was treated at passage 4). Three biological replicates were used for each time point, and each measurement was done in triplicate. Error bars represent means ± SEM. Two-tailed t-tests (α = *0.05)* were performed for each data point compared to *RASD1* mRNA expression at 0 h of Dex treatment. ∗*P* < 0.05; ∗∗*P* < 0.01; ∗∗∗*P* < 0.001.

We found that *RASD1* expression may be donor-, and specifically age-, dependent (Fig. 2). Dex regulated *RASD1* expression in a time-dependent fashion in the TM cells from donors 1–5, with a 1.4- to 5.6-fold increase of *RASD1* mRNA expression within 30 min to 1 h compared to the Veh treatment, followed by gradual decline within 3 h. One exception was found in the TM cells from donor 5, where *RASD1* mRNA expression significantly increased again at time point 2 h and declined at time point 3 h of Dex treatment. In contrast, for donors 7–9, *RASD1* mRNA level exhibited no significant change with 0–3 h of Dex treatment, while donor 6 demonstrated a significant downregulation of *RASD1* mRNA at 1 h of Dex treatment. We then characterize donors 1–5 as the responders and donors 6–9 as the non-responders based on the differing response of *RASD1* expression upon 0–3 h of Dex treatment. Moreover, the ages of the responders ranged from 18 to 19 (donors 1–2) and 59–66 (donors 3–5), while the ages of the non-responders (donors 6–9) ranged from 38 to 55. These data suggest that *RASD1* expression could be regulated in an age-dependent fashion, with rapid and robust elevation in younger- and older-aged individuals after GC treatment. In contrast, middle-aged individuals may not exhibit the same response.

The findings of this study reveal that Dexras1 is regulated in a timely manner by dexamethasone, with rapid and robust upregulation at 30 min to 1 h after dexamethasone treatment in human TM cells from younger- and older-aged donors. This age-related pattern of Dexras1 responders vs. non-responders to steroid treatment reflects the clinically observed pattern of steroid response. Steroid response is the phenomenon of IOP elevation induced by the use of corticosteroids and is thought to occur due to pathophysiologic changes in the TM that lead to outflow dysfunction. Studies estimate that around 35 % of the general population develops clinically significant steroid-induced IOP elevation, and age has been identified as a key risk factor for steroid response. Young children seem particularly susceptible to the complications of steroid usage. Clinical studies have reported IOP elevation within a few days of initiating topical steroid treatment in children, as opposed to weeks of treatment in adults, with as many as 80 % experiencing an IOP increase of 15 mmHg or more [14]. Consequently, steroid-induced glaucoma in the pediatric population can occur earlier and progress more rapidly than in adults, though the underlying mechanisms behind the more pronounced steroid response in children remain unclear. On the other hand, older age is also a risk factor for steroid-induced glaucoma [15], presumably due to a decrease in overall number of trabeculocytes with age and pre-existing age-related TM dysfunction that is further impaired by the use of corticosteroids.

A potential mechanism by which induction of Dexras1 may contribute to IOP elevation and glaucomatous pathology is GC-associated adipogenesis. Previous research has indicated that Dexras1 plays a pivotal role in adipogenesis, the process of forming adipocytes. Cha et al. demonstrated that overexpression of Dexras1 induces adipogenesis, while depletion of Dexras1 inhibits the adipogenic differentiation of 3T3-L1 cells, a fibroblast line considered to be the best-characterized model of adipogenesis [16]. Deficiency of Dexras1 has since also been associated with reduced adipogenesis [17]. Abnormal fat deposits in the eye may disrupt the TM, leading to obstruction of the conventional aqueous humor outflow pathway, which can lead to an elevation in IOP.

Adipogenesis in the eye may also lead to aberrations in IOP control due to vascular dysfunction. Adipocytes secrete adipokines, a diverse group of biomolecules involved in a variety of physiological processes such as regulating metabolism, inflammation, and cardiovascular health [18,19]. Leptin and adiponectin are examples of well-known adipokines, and systemic elevation in levels of these adipokines is known to increase blood pressure and heart rate, promoting adverse effects on cardiovascular function [18,20,21]. Local secretion of adipokines in the eye are believed to exert the same effect on the blood vessels, causing blood vessel constriction and thus elevating IOP. Adipose tissue is also known to secrete proinflammatory cytokines, such as tumor necrosis factor-alpha (TNF-alpha) and interleukin-6 (IL-6), resulting in chronic low-grade inflammation that can cause blood vessel impairment, increased oxidative stress, and endothelial dysfunction, all of which can contribute to ocular hypertension [22,23].

In conclusion, this study illustrates a spectrum of Dexras1 response in TM cells among donors of different ages, with a greater induction of *RASD1* expression observed in the cells of younger and older donors. This finding may explain the increased susceptibility of younger and older-aged individuals to SIOH. Our study also establishes that Dexras1 is regulated by GC treatment in TM cells in a timely manner. We hypothesize a promising IOP regulation model in which GC-induced Dexras1 activation leads to adipogenesis in the eye. Excess adipogenesis in the eye may result in secretion of adipokines that promote localized inflammation detrimental to blood vessel health, and direct obstruction of the TM by fat molecules that reduce aqueous humor outflow, ultimately resulting in elevated IOP. Future research should aim to elucidate the mechanisms by which GC induced Dexras1 expression may alter TM cell function and the interconnection between Dexras1 and other signaling pathways.

## CRediT authorship contribution statement

**ChihWei Chen:** Writing – review & editing, Writing – original draft, Formal analysis, Data curation. **Jiapeng Han:** Writing – review & editing, Writing – original draft, Formal analysis, Data curation. **Luis Sanchez:** Writing – original draft, Validation, Supervision, Formal analysis, Data curation. **Judy L. Chen:** Writing – review & editing, Supervision. **Jie J. Zheng:** Writing – review & editing, Supervision, Software, Resources, Project administration, Methodology, Funding acquisition, Formal analysis, Conceptualization.

## Declaration of competing interest

The authors declare that they have no known competing financial interests or personal relationships that could have appeared to influence the work reported in this paper.
