# Supplementary material for: Characterizing the expression profile of Dexras1 in human trabecular meshwork cells
Source: Biochem Biophys Rep. 2025 Jun 10;43:102077. doi: 10.1016/j.bbrep.2025.102077 (PMC12182328; doi:10.1016/j.bbrep.2025.102077)
Supplement: Multimedia component 1 [file mmc1.docx]

**Supplemental Materials**

| Donor | Tissue # | Age | Sex |
| --- | --- | --- | --- |
| 1 | TM337 P3 | 19 | NA |
| 2 | TM306 P4 | 18 | Female |
| 3 | TM287A P3 | 59 | NA |
| 4 | TM401 P3 | 61 | NA |
| 5 | TM286B P3 | 66 | Male |
| 6 | TM349 P3 | 38 | Male |
| 7 | TM298C P3 | 40 | NA |
| 8 | TM346A P3 | 55 | NA |
| 9 | TM345D P3 | 50 | NA |

Table S1: A summary of the age and sex information of the 9 TM tissue donors in this study. NA: not available.
